# Chemoprotective Effect of Sobatum against Lithium-Induced Oxidative Damage in Rats

**DOI:** 10.4103/0975-1483.62217

**Published:** 2010

**Authors:** K Vijaimohan, J Mallika, Devi CS Shyamala

**Affiliations:** *Department of Biochemistry, University of Madras, Chennai - 600 025, India*; 1 *Department of Biomedical Engineering, SSN College of Engineering, Chennai - 603 110, India*

**Keywords:** Antioxidants, lithium toxicity, oxidative damage, sobatum, *Solanum trilobatum*

## Abstract

Lithium therapy mainly used in curing some psychiatric diseases responsible for numerous undesirable side effects on different organs in humans. The present study explores the beneficial effect of sobatum, a purified compound of *Solanum trilobatum*, on lithium carbonate (Li_2_CO_3_)-induced multiple organ toxicity in rats. Li_2_CO_3_ (150 mg/kg body weight) was administered orally in drinking water for a period of 30 days to induce toxicity in rats. Li_2_CO_3_ could induce lipid peroxidation to a significant extent that was accompanied by marked reduction in reduced glutathione, SOD, CAT, GST, GPX activities, and parallel decline in ATP in tissues. Toxicity resulted in abnormal elevation of lipids such as cholesterol, triglycerides, phospholipids, and fatty acids in liver tissues. Treatment with sobatum affords substantial protection in liver and heart by altering all the parameters to near normal levels that were further confirmed by histological examination. Sobatum prevents Li_2_CO_3_-induced oxidative damage of DNA by reducing DNA fragmentation indicating its block on cell death. However, these results demonstrated that sobatum has the ability to suppress the drug-induced toxicity.

## INTRODUCTION

Lithium is widely used in the treatment of bipolar disorders, refractory depressive disorders, and in the long-term prophylaxis of cluster headache. It has a narrow therapeutic index. Chronic lithium intoxication is more common. There is gradual accumulation of lithium, usually due to decreased excretion. Lithium poisoning causes impaired renal function, drug interactions, volume depletion, and concurrent illnesses such as congestive heart failure or cirrhosis.[1,2] Lithium exerts its effects on a wide range of cellular functions by inhibiting inositol production, affects the protein kinase C signaling pathway, and inhibits glycogen synthase kinase 3 (GSK3).[3] Early recognition and management of lithium-induced organ toxicity can save lives and reduce significant morbidity.

In the Ayurvedic system of medicine, *Solanum trilobatum* L. (Solanaceae) (common name: Tuduvalai) have been used for respiratory, cardiac, liver, renal, and cancer diseases.[4] Sobatum, alpha-solamarine, solanine, solasodine, glycoalkaloid, diosogenin, and tomatidine are the constituents isolated from this plant.[5] Previous studies in our lab proved that treatment with *S. trilobatum* extract enhance the recovery from carbon tetrachloride-induced hepatic damage in rats.[6] It was also reported that *S. trilobatum* possesses anti-inflammatory, anti-ulcerogenic, and antimicrobial activities.[7,8] In our laboratory, sobatum has been isolated and purified to homogeneity from herb *S. trilobatum* and believed to be an active principle responsible for its beneficial action on vital organs. Earlier studies proved that sobatum has the antitumor property and produce no toxicities.[9] Sobatum also possesses anti-inflammatory and free radical scavenging activities by inhibiting superoxide radical production.[10,11] These points made us interested to do research on sobatum against lithium-induced toxicity. However, no studies have been performed to study whether sobatum have beneficial effects on lithium carbonate (Li_2_CO_3_)-induced toxicity in experimental models of organ injury, which may have implications in managing humans with accidental exposures to such compounds.

Considering above information, the present study aims to investigate the effect of sobatum on toxicity caused by Li_2_CO_3_ in liver, heart, and kidney tissues of experimental rats through evaluating lipid peroxidative content, hepatic lipid levels, antioxidant enzymes, adenosine triphosphate (ATP) levels, apoptotic cell death, and histological changes.

## MATERIALS AND METHODS

### Chemicals

All chemicals and reagents were of analytical grade or of highest purity, commercially available. Lithium carbonate, glutathione, disodium phenyl phosphate, and bovine serum albumin were purchased from Sigma Chemical Co., St Louis, MO, USA. EDTA, DNAase, and proteinase K were purchased from British Drug House Pvt. Ltd Glaxo Division, Mumbai, India.

### Plant materials and extraction of sobatum

The whole plant of *Solanum trilobatum* was collected in and around Chennai, India, and authenticated in the Department of Pharmacognosy, Captain Srinivasa Murthi Drug Research Institute for Ayurveda, Chennai, India. A voucher specimen has been deposited in the same institute (No: 1785). The plant samples were shade-dried and powdered using an electric blending machine. For the preparation of the extract, 100 g of powdered plant material was subjected to extraction with 90% petroleum ether in a Soxhlet apparatus up to four cycles. After filtration through Whatman filter paper no. 40, the filtrate was dried using a lyophilizer.

The petroleum ether extract of *S. trilobatum* was subjected to chromatography using Spherisorb-ODS2 25 cm × 4.6 cm, 5 cm, C_18_ silica column using water and petroleum ether/ethyl acetate (75:25) as a solvent at a flow rate of 1 ml/min. These fractions were concentrated and crystallized from solvent giving only one pure crystalline compound that was identified and compared with authentic sample. For oral administration, the sobatum compound was dissolved in 0.5 ml of 10% dimethyl sulphoxide (DMSO) immediately before use.

### Animals

Adult male albino rats of Wistar strain weighing about 150-200 g were purchased from Fredrick Institute, Padapai, India. The animals were housed at 27 ± 2°C temperature, 55% humidity, and a 12 h-light/dark cycle. They were fed with standard laboratory chow (Hindustan Lever Foods, Bangalore, India) and provided with water *ad libitum*. Experimental protocols were approved by our Institute ethical committee, which follows the guidelines of Institutional Animals Ethics Committee (IAEC) (360/01/a/CPSEA).

### Experimental design

Animals were divided into four groups each containing six animals. Group I animals served as control who received the vehicle (0.5 ml of 10% DMSO) alone orally. Group II animals constituted the toxicity group, who received Li_2_CO_3_ (150 mg/kg body weight) orally in drinking water for a period of 30 days.[12] Group III animals received Li_2_CO_3_ (150 mg/kg body weight) orally in drinking water for a period of 30 days and treated simultaneously with sobatum orally at doses (30 mg/kg body weight/day)[13] dissolved in 0.5 ml of 10% DMSO. Group IV rats received sobatum orally at doses (30 mg/kg body weight/day) dissolved in 0.5 ml of 10% DMSO.

After 24 h of sobatum last dose treatment, animals were sacrificed by cervical decapitation under pentobarbitone sodium (60 mg/kg) anesthesia and blood was collected without an anticoagulant to separate serum for biochemical investigation. The liver and heart organ were dissected out immediately and washed in ice-cold saline. The tissues were then homogenized in ice-cold Tris-HCl buffer (0.1 M, pH 7.4) and 10% homogenate was obtained and used for the following biochemical investigations.

### Determination of lipid peroxidation and reduced glutathione levels

LPO was measured in hepatic homogenates according to the method of Okhawa *et al*. based on the formation of thiobarbituric acid reactive substances (TBARS) and expressed as the extent of malondialdehyde (MDA) production.[14]

Glutathione (GSH) levels were quantified by the method of Moron *et al*.(1979).[15] The tissue homogenate was treated with 1 ml of 5% TCA in 1 mM EDTA and centrifuged at 2000 g for 10 min. After that 1 ml of the filtrate was mixed with 5 ml of 0.1 M phosphate buffer (pH 8.0), and 0.4 ml of 5,5’-dithiobis (2-nitrobenzoic acid). The absorbance of the solutions was estimated at 412 nm against blank. The level of ATP in the heart tissue was determined by the method of Ryder.[16]

### Estimation of antioxidant enzymes

The activity of superoxide dismutase (SOD) was measured, using an assay based on the ability of SOD to inhibit the autoxidation of pyrogallol by 50%. One unit of enzyme is defined as the amount of enzyme that causes half maximal inhibition of pyrogallol autooxidation/mg protein/min.[17] The assay of catalase was performed by following the method of Aebi. One unit of enzyme activity is defined as the amount of enzyme required to decomposed 1 mmol of H_2_O_2_/mg protein/min.[18] The activity of glutathione peroxidase (GPx) was measured using a coupled enzyme assay as described by Lawrence and Burk.[19]

### Estimation of lipid profile

Lipid was extracted from liver tissues by the method of Folch *et al*. using the chloroform-methanol mixture (2:1 v/v).[20] The total cholesterol, free cholesterol, free fatty acids (FFA), triglyceride (TG), phospholipids (PL) after perchloric acid digestion have been determined in liver tissues of sobatum-treated rats.[21–25]

### Histopathology

The heart tissue samples were preserved in 10% neutral buffered formalin for 24 h, processed for routine paraffin block preparation, sections of 5μm thick were cut and stained with hematoxylin and eosin. These were examined under the light microscope for histological changes in tissues.

### Studies on DNA damage

The kidney tissue was used for the isolation of DNA by homogenizing in PBS-E (50 mM sodium phosphate buffer containing 0.9% saline and 20 mM EDTA, pH 8) and suspended in 2 ml of PBS-E containing 0.5 mg/ ml of collagenase. The suspension was incubated at 37°C for 1 h with stirring, followed by the addition of pronase E (1 mg ml), and further incubated for 15 min at 37°C. It was then centrifuged at 1000 rpm for 5 min. The pellet was dispersed and incubated with 2 ml of a lysis buffer containing 50 mM Tris-HCl^−^, pH 8, 20 mM EDTA, 10 mM NaCl and 1% w/v SDS for 15 min. It was centrifuged again at 14,000 × *g* for 15 min, and DNA was isolated from the lysate by a phenol-chloroform extraction procedure.[26] DNA was dissolved in 10 mM Tris-HCl^−^, pH 8, containing 1 mM EDTA by gentle shaking at 65°C. Residual contaminating RNA was removed by incubating the DNA solution with 1 μg/ml DNase-free RNase at 37°C for 1 h followed by 0.1 mg ml proteinase K for 3 h. Phenol-chloroform extraction was repeated to obtain purified DNA that was dissolved in 10 mM Tris-HCl^2^ buffer, pH 8, containing 1 mM EDTA. To study DNA fragmentation, DNA was loaded on to a 1.5% agarose gel. In addition to compare the test samples with the standard antioxidant 1 μg of catalase was used and electrophoresis was carried out at 100 V for 1.5 h in TBE (90 mM Tris borate, 2 mM EDTA, pH 8) buffer and DNA was visualized by UV exposure after staining with ethidium bromide.

### Statistical analysis

Data are expressed as mean ± SD. The results were computed statistically (SPSS software package, version 11.0) using one-way analysis of variance. *Post hoc* testing was performed for inter-group comparison using the Dunnett’s ‘*t*’ test. *P* < 0.001, *P* < 0.01, *P* < 0.05 were considered to be statistically significant.

## RESULTS

Compared with controls, lithium-toxicated rats were accompanied by increased TBARS production and decreased antioxidant defense enzyme activities, suggesting oxidative stress in liver tissue [Table 1]. Administration of sobatum led to a marked reduction in the level of TBARS and significant enhancement in antioxidant defense enzyme activities as compared to lithium-treated rats. On the other hand, in sobatum-alone-treated rats, no significant change in enzyme activities could be observed, as compared to that of normal control.

**Table 1:** Effect of sobatum on the changes of liverantioxidant defense enzyme activities in Li_2_CO_3_ administered rats

Groups	SOD	CAT	GPx	TBARS	GSH
I	8.61±0.45	4.81±0.19	0.02±0.001	0.56±0.04	5.98±0.03
II	6.51±0.32*	2.98±0.15*	0.05±0.003*	1.02±0.09*	3.99±0.02*
III	8.17±0.35^a^	4.01±0.23^a^	0.04±0.002^a^	0.70±0.05^a^	4.82±0.03^a^
IV	8.64±0.41	4.83±0.31	0.02±0.001	0.54±0.04	6.01±0.05

Results are expressed as mean±SD for groups of six animals each; SOD: Superoxide dismutase; CAT: Catalase; GPx- Glutathione peroxidase: U/mg protein. TBARS-nmoles/mg protein; GSH-reduced glutathione - μg of GSH/mg protein

Values are expressed as significant difference at *P <0.001 as compared with control group

significant difference at ^a^*P* <0.001 as compared with Li_2_CO_3_ alone administered rats

**Figure 1:**
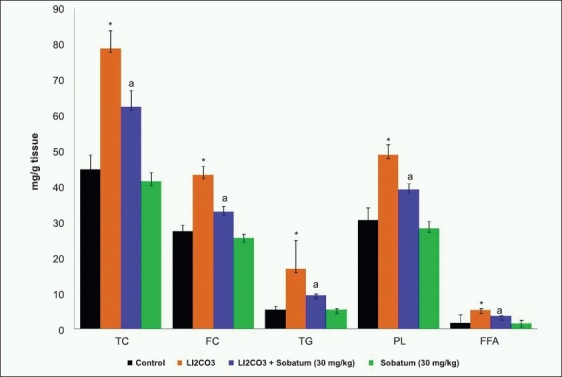
Effect of sobatum on the levels of total cholesterol (TC), free cholesterol (FC), triglyceride (TG), phospholipid (PL) and free fatty acid (FFA) in the liver tissue of Li_2_CO_3_ administered rats. Results are expressed as mean ±SD for groups of six animals each. *P<0.001 as compared to control; ^a^P<0.01 as compared to Li_2_CO_3_ alone administered rats

Li_2_CO_3_ caused a significant increase in free cholesterol, total cholesterol, triglycerides, phospholipds, and fatty acid content as compared to the corresponding control group. Sobatum treatment showed a marked protection against this enhanced lipid levels in the liver tissue of Group II rats [Figure 1]. No significant differences were observed between normal and sobatum-treated rats. Lipid levels and other biochemical parameters were not altered upon sobatum treatment.

**Figure 2:**
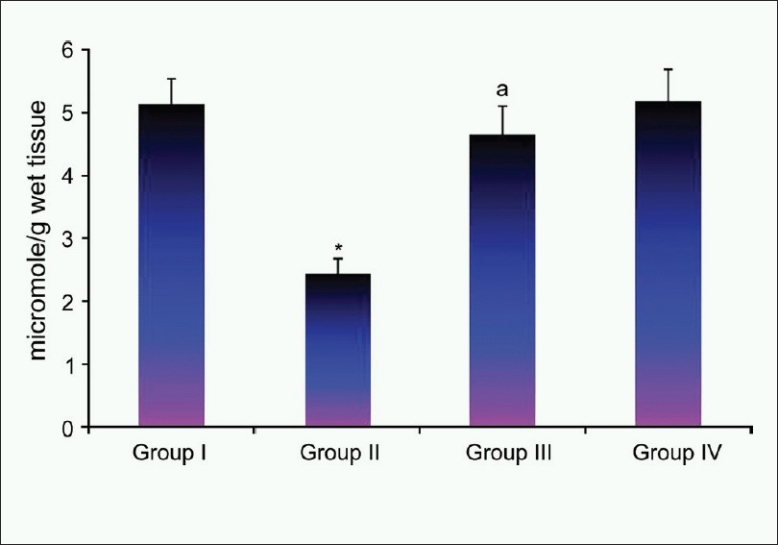
Effect of sobatum level of adenosine triphosphate in heart tissue of control and experimental rats

Figure 2 shows the level of myocardial ATP content in normal and experimental groups of rats. Significant reduction in the level of ATP content in the heart tissue of lithium-induced rats as compared to that of Group I rats. The administration of sobatum maintained the level of ATP at near normalcy in Group III rats as compared to that of Group II rats, reflecting its ability to maintain the function of the heart mitochondria at near normal status.

**Figure 3:**
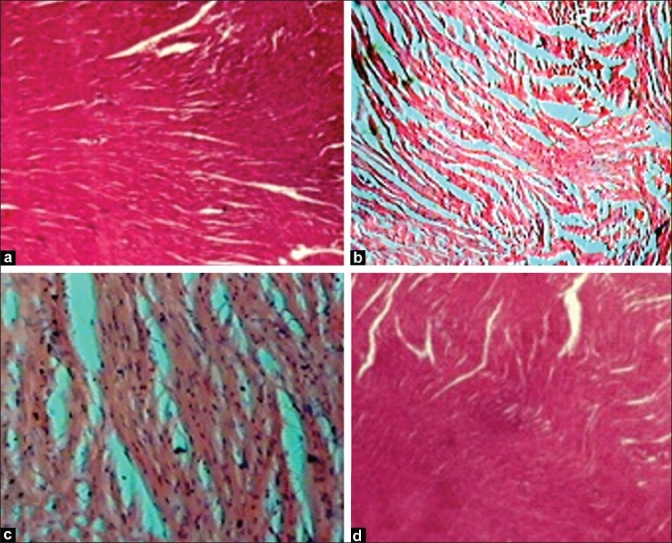
Sections of heart tissue obtained from rats of control groups and treated with sobatum in lithium carbonate-induced rats. (H and E, ×100)

Microscopic examination of heart tissue of the Group I control rats showed normal myocardial fibers and muscle bundles with normal architecture. Heart tissue of Group II rats showed separation of myocardial fibers with inflammatory mononuclear collections, edema, and myocardial necrosis. Sobatum-alone-treated Group IV rats showed normal myocardial fibers with no pathological changes. Myocardial section of sobatum-treated Group III rats showed slightly separated myocardial fibers with small focus of inflammatory mononuclear collections with the absence of necrotic damage [Figure 3].

**Figure 4:**
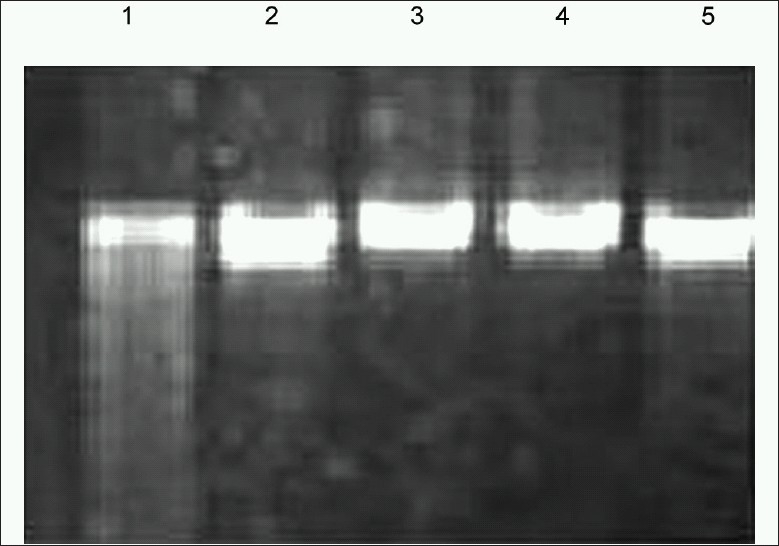
Effect of sobatum on lithium carbonate-induced DNA fragmentation in rats

Agarose gel electrophoresis of kidney tissue DNA, treated with or without sobatum in Li_2_CO_3_-treated rats is shown in Figure 4. Control rat and sobatum-treated rat showed the absence of DNA fragmentation in kidney tissue, which was observed in Lane 3 and Lane 4. In Lane 1, the toxic effects of Li_2_CO_3_ were reflected in a significantly higher rate of apoptosis in kidney tissue of Group II rats so no DNA band was observed. However, in Lane 2, Li_2_CO_3_-induced DNA fragmentation was markedly reduced by treatment with sobatum suggesting its role to prevent cell death. Sobatum provides significant protection against DNA damage in comparison with the already known antioxidant, catalase (Lane 5) suggesting its antiapoptotic role during oxidative damage.

## DISCUSSION

Lithium toxicity represents a state of increased oxidative stress, which is mainly based on the evidence of increased lipid peroxidation (LPO), or by indirect evidence of reduced antioxidant reserve, such as SOD and catalase enzymes, in animal models.[27] Sobatum have been reported to play a beneficial role in the reduction of free radical production.[11] In our present study, the administration of sobatum exerted beneficial effects against lithium toxicity followed by the fall in lipid peroxidation products. During physiological states, SOD metabolizes superoxide anion (O_2_^−^), producing hydrogen peroxide (H_2_O_2_), which can react with iron to generate highly reactant hydroxyl radicals via the Fenton reaction. CAT is the most important peroxidase in detoxifying excess hydrogen peroxide to prevent hydroxyl production. Thus an increase in SOD or CAT levels *per se* does not necessarily indicate increased oxidative stress, whereas an imbalance between SOD and CAT activities could lead to an excessive generation of free radicals.[28] The antioxidative defense system like SOD, CAT, and GPx showed lower activities in liver during toxicity condition. Treatment of the lithium-induced animals with sobatum restored the altered activities of SOD, CAT, and GPx. This study suggests that possible mechanism of this protective activity against lithium toxicity may be due to free radical-scavenging and antioxidant activities.[5]

Lithium-induced elevation in lipid levels could be due to increase in biosynthesis and decrease in its utilization. Li_2_CO_3_ induces free radicals, which may cause cellular cholesterol accumulation, by increasing cholesterol biosynthesis and its esterification. The significant increase in the rate of TG, PL, and FFA synthesis by the liver is a well-known risk of toxicity caused by Li_2_CO_3_.[29] Abnormal activities of lipid-metabolizing enzymes contributed to these hyperlipid changes in liver caused by Li_2_CO_3_. *S. trilobatum* is a hypolipidemic agent, which reduces the rise in total cholesterol, free cholesterol, TG, FFA, and PL.[30] In addition, antioxidants such as sobatum could be beneficial in preventing the increase in lipidemic status, suggesting that it would prevent the toxicity of Li_2_CO_3_ on lipid metabolism.

In the heart, ATP is synthesized mainly in the mitochondria through oxidative phosphorylation and transported to the contractile apparatus, where it is consumed by myosin ATPase to generate force. The viability of the cell depends for the most part on the integrity of several vital subcellular systems, and the major source of energy for contraction comes from the oxidative metabolism of mitochondria in the cell. In the present study, administration of sobatum maintained the level of ATP at near normalcy in Group III rats as compared to that of lithium-induced rats, reflecting its ability to maintain the function of the heart mitochondria at near normal status. Many recent evidences indicated that mainly heart damage may result from Li_2_CO_3_ intoxication[31] which was evident in the present study by histopathologic findings. Protection against heart damage in sobatum-treated rats showed normal functional cytoarchitecture of heart tissues.

Involvement of ROS, oxidative damage of DNA, and DNA fragmentation has also been evident in apoptotic cell death in renal injury.[32] Cytotoxic effects of Li _2_CO_3_ that affect the liver tissue are manifested by the disturbances of NO, a key mediator of signaling events linked to apoptotic cell death.[33] Sobatum prevents Li_2_CO_3_-induced DNA fragmentation, suggesting its antiapoptotic role to block cell death during damage. Sobatum could have a unique capacity to block this oxidative damage similar to that shown by H_2_O_2_ scavenger, catalase, indicating its potent antioxidant role to protect DNA from the attack of ROS.

## CONCLUSION

The results of the present study indicate that administration of sobatum attenuates lithium-induced organ toxicity in rats through counteraction of free radicals by its antioxidant property, antiapoptotic effect by blocking DNA fragmentation and attenuation of toxic changes in vital organs.
